# Improving Evidence-Synthesis for School-Based Obesity Prevention Interventions. Comment on Ginell et al. Unreliable Findings Due to Miscalculations and Errors. Comment on “Nally et al. The Effectiveness of School-Based Interventions on Obesity-Related Behaviours in Primary School Children: A Systematic Review and Meta-Analysis of Randomised Controlled Trials. *Children* 2021, *8*, 489”

**DOI:** 10.3390/children11080959

**Published:** 2024-08-09

**Authors:** Vanessa A. Shrewsbury, Rachael M. Taylor, Tammie Jakstas, Angeliek Verdonschot, Clare E. Collins

**Affiliations:** 1 School of Health Sciences, College of Health, Medicine and Wellbeing, University of Newcastle, Callaghan, NSW 2308, Australia; rachael.taylor@newcastle.edu.au (R.M.T.); tammie.jakstas@uon.edu.au (T.J.); clare.collins@newcastle.edu.au (C.E.C.); 2 Food and Nutrition Research Program, Hunter Medical Research Institute, New Lambton Heights, NSW 2305, Australia; angeliek.verdonschot@newcastle.edu.au; 3 School of Education, College of Human and Social Futures, University of Newcastle, Callaghan, NSW 2308, Australia; 4 Centre for Active Living and Learning, College of Human and Social Futures, University of Newcastle, Callaghan, NSW 2308, Australia

As researchers with substantial experience in the child obesity field [1] and school-based interventions [2], we endeavour to stay abreast of the current evidence on effective prevention and treatment strategies in the school setting. Of interest is Ginell et al.’s [3] re-analysis of Nally et al.’s [4] systematic review and meta-analysis on the effectiveness of school-based interventions in changing body mass index (BMI) and obesity-related behaviours in primary school children. Ginell reported “no evidence indicating significant effects for the mean difference in BMI between intervention and control groups” and called for retraction of Nally’s paper [3].

Umbrella reviews are systematic collections and assessments of multiple systematic reviews and meta-analyses [5]. With over one billion primary and secondary students collectively worldwide [6], of whom 390 million are already affected by overweight or obesity [7], umbrella reviews provide much needed, high-level, evidence synthesis for policy makers to plan school-based obesity prevention initiatives. We have identified two umbrella reviews which exclusively focus on behaviour, weight and wellbeing-related effects of interventions in school settings, for preventing or treating overweight or obesity (Amini et al., 2015; and Safron et al., 2011) [8,9], but these are now out of date.

Rozga & Handu’s umbrella review published in 2023 has somewhat updated the evidence in this field, by examining obesity prevention interventions including a nutrition component for 2–17 year olds, across a range of settings including schools, on BMI measures, overweight/obesity prevalence and incidence, and cost-effectiveness [10]. Whilst we noted Nally et al.’s [4] data were included in Rozga & Handu’s umbrella review, there was no mention of Ginell et al.’s re-analysis [3]. We also identify examples of other relevant systematic reviews published within the last five years, including Hodder et al. [11] in 2022 and Liu et al. [12] in 2019, and there may be other reviews, which are yet to be incorporated in an umbrella review in this field.

It is essential that policy makers for school-based obesity prevention initiatives globally receive timely access to accurate, up-to-date, regionally and culturally relevant research evidence synthesis, to inform policy and intervention decisions. To facilitate this, we recommend:

- **Publication and maintenance of high-quality *living* reviews**: Living systematic reviews are defined as “a systematic review that is continually updated, incorporating relevant new evidence as it becomes available” [13]. Methodological and practical guidance for conducting living systematic reviews is available from Cochrane [14] and other sources [15]. One of the potential advantages of living systematic reviews, over traditional systematic reviews, is that identified errors or limitations can be addressed and assimilated into evidence syntheses and meta-analyses in a more timely and co-ordinated manner. We speculate that in the not-too-distant future, guidance for living umbrella reviews will also be available, as this type of evidence is already being published [16].
- **Publication of high quality systematic and umbrella reviews**: If it is not practical or feasible to produce high-quality *living* reviews, then it is imperative that high quality traditional systematic and umbrella reviews are undertaken. In addition to current guidelines and reporting standards for producing high quality systematic [17] and umbrella reviews [5], a recently published living systematic review has identified and discussed common flaws in published reviews, to improve understanding of problems in the conduct, methods and reporting [18].
- **Careful consideration of nuances in school-based obesity prevention interventions data when conducting reviews**:
  1. For example, while Nally et al. [4] performed some subgroup analyses (e.g., intervention duration, presence of underpinning theory, single versus multicomponent interventions), other similar reviews have performed subgroup analyses based on additional potentially moderating factors including age-group, geographical locality, and specific types of intervention components [11]. In future umbrella reviews, the subgroup analyses conducted in systematic reviews/primary studies also need to be carefully considered to help with accurately interpreting findings.
  2. Another example, as Amini et al.’s [8] umbrella review highlights, is that few school-based obesity interventions report on potential differential effects on outcomes, according to weight status at baseline. As this is an important potential moderator that needs to be evaluated, authors of primary studies in this field also have a role to play in reporting intervention effects by subgroups, including baseline weight category, when possible.

In concluding this article, we acknowledge the complexities of conducting and interpreting school-based obesity prevention reviews. We provide readers with a collation of recent publications that can inform rigor in evidence-synthesis for school-based obesity prevention interventions.

## Data Availability

No new data were created or analyzed in this study. Data sharing is not applicable to this article.
